# Supplementary figures and images for: Plastics in Porifera: The occurrence of potential microplastics in marine sponges and seawater from Bocas del Toro, Panamá
Source: PeerJ. 2021 Jul 8;9:e11638. doi: 10.7717/peerj.11638 (PMC8272925; doi:10.7717/peerj.11638)

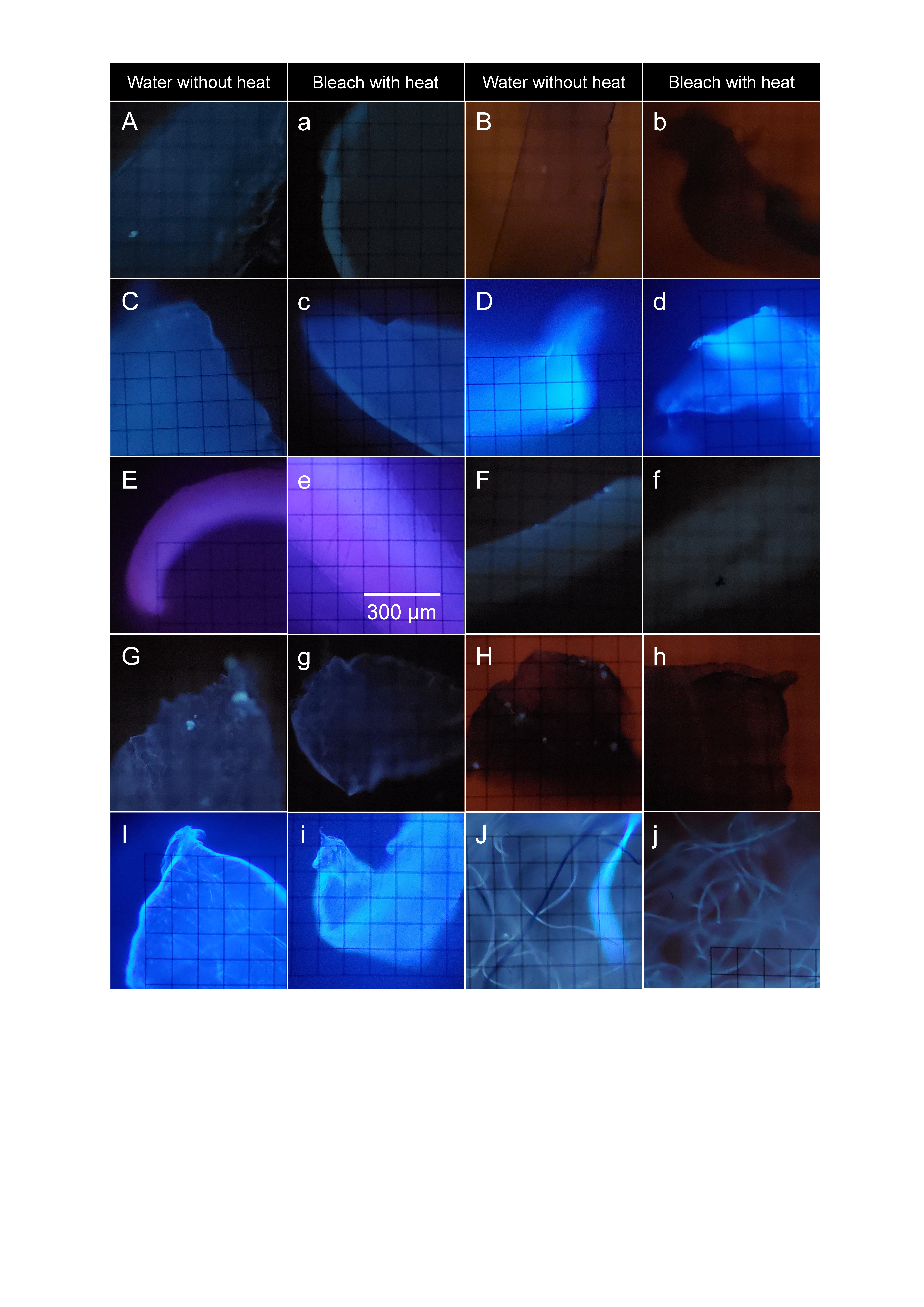

Supplement: Supplemental Information 1 — (A, a) white HDPE. (B, b) brown HDPE. (C, c) yellow PP. (D, d) white PP. (E, e) red PP. (F, f) white PVC. (G, g) white unknown (recycling label #7). (H, h) brown unknown. (I, i) white PETE. (J, j) polyester. Capital letters indicate plastics that were added to MilliQ and were not heated, whereas lowercase letters indicate plastics that were bleached and heated. Note the minimum effect of bleach and heat on physical integrity and fluorescence behavior. Images were taken at 100× total magnification and the scale bar shown in “e” is applicable to all images. [file peerj-09-11638-s001.png]
